# Supplementary material for: Are carbon emissions trading and green financial instruments synergistic? -Comprehensive quantitative research based on content analysis
Source: PLoS One. 2024 Mar 7;19(3):e0298601. doi: 10.1371/journal.pone.0298601 (PMC10919601; doi:10.1371/journal.pone.0298601)
Supplement: S1 Data — (ZIP) [file pone.0298601.s001.zip › Data/地级市Z_F工作报告.zip!/╣π╢1⁄2/│▒╓▌╩╨/└┤╫╘╣1⁄2╓┌║┼í╛┬φ┐╦╩2╛▌═°í┐_2010.docx]

各位代表：

现在，我代表市人民政府向大会作工作报告，请予审议，并请市政协委员和其他列席人员提出意见。

2009年工作回顾

2009年，是我市积极应对国际金融危机、努力保持经济平稳较快发展，并取得显著成绩的一年。过去一年，面对严峻复杂、曲折多变的经济形势，在省委省政府和市委的正确领导下，在市人大、市政协的监督支持下，市政府以科学发展观为指导，以“落实推进年”活动为动力，全面贯彻落实中央保增长、保民生、保稳定的决策部署，按照省委省政府“三促进一保持”的要求，全力以赴保增长，抓住时机扩内需，千方百计稳外贸，着眼未来促调整，扎扎实实惠民生，有效地促进了经济社会全面发展，圆满完成了市十三届人大四次会议确定的各项目标任务。

一、经济发展水平稳步提高

经济平稳较快增长。全市实现生产总值480.2亿元，比上年增长12.5%，比全省平均水平高出3个百分点，增幅在全省排名由上年的第9位上升为第7位。其中，第一、第二、第三产业增加值分别增长5.9%、10.7%和16.5%。人均生产总值1.87万元，增长11.8%。

财政收支情况良好。税收征管力度加大，财税收入平稳增长。地方财政一般预算收入18.25亿元，增长13.9%。其中，非税收入1.76亿元，增长39.7%。各项税收总额45.68亿元，增长7.8%。其中：国税国内收入29.67亿元，增长4.8%；地税收入16亿元，增长13.8%。地方财政一般预算支出44.3亿元，增长18.4%。

人民生活有新提高。市区居民人均可支配收入12398元，增长9.5%；农民人均纯收入5492元，增长10.4%。汽车下乡、家电下乡和以旧换新等鼓励消费政策得到落实，建成家电下乡网点近200个，家电、商品房和汽车等消费旺盛。全社会消费品零售总额210.63亿元，增长23.4%。居民消费价格指数下降3%。

二、工业在调整中优化发展

产业规模发展壮大。全市工业增加值252亿元，增长10.3%。其中，规模以上工业增加值149.4亿元，增长13.6%，增速居全省第10位。全市新增工业投资86.3亿元，增长13.2%；新增规模以上工业企业203家，累计1185家；新增产值超亿元企业11家，累计103家；新增私营企业1350家、个体工商户7460户，个体户转型登记为工商企业960家。

产业竞争能力增强。企业技术改造步伐加快，年度181个技改项目顺利实施，完成技改投资22.1亿元，增长72%。获得国家和省立项的科技项目82项，省市共建陶瓷先进制造业基地的135个项目实施情况良好。新增省工程中心2家、省企业技术中心6家、省专业镇1个，累计达到20家、15家和15个。新获得中国驰名商标1件，获得省级以上名牌名标累计达到339项。企业主导或参与技术标准制修订4项，累计已有47家企业参与30个国家标准、11个地方标准和24个行业标准的制订和修订。国家陶瓷及水暖卫浴产品质检中心通过验收，全市已建成省级以上质检机构4家。知识产权保护工作得到加强，专利申请量和授权量均居全省第9位。

产业结构调整优化。陶瓷、服装等八大特色产业保持发展，饶平黄冈水族机电被评为省产业集群升级示范区，潮州工艺服装（婚纱晚礼服）、饶平水产被认定为省特色产业基地。高新技术产业逐步发展，新增高新技术企业12家、累计30家，新增省级民科企业5家、累计153家。临港产业项目建设不断推进,大唐电厂3、4号机组完成试运行，临港工业增加值17.8亿元，增长12%。规模以上重工业增加值60.68亿元，增长15.3%，增幅比轻工业高出2.9个百分点。

服务保障工作得到加强。积极实施“中小企业解困帮扶工程”，出台促进中小企业发展30条措施。财政专项用于企业科技创新、市场开拓等方面的资金投入2210万元，比增22.7%。大力推进银企协作，强化信贷支持，全市金融机构各项存款余额556.3亿元、贷款余额192.7亿元，分别增长16.7%和20.4%。加强融资担保体系建设，组建市金山投资开发公司、市潮发担保公司、潮安县兆丰小额贷款公司等机构。支持鼓励企业上市融资，指导帮助三环、长城等企业申报上市。

三、“三农”工作成效明显

农业发展水平稳步提高。粮食生产稳定增长，播种面积65.9万亩、总产26.6万吨，分别增加0.7万亩和1.87万吨。农业生产示范基地和现代农业园区建设加快，规模化、产业化、标准化水平提高。茶叶、潮州柑、花卉等特色农业继续发展。“潮州柑”地理标志证明商标成功注册，“凤凰单丛茶”地理标志产品保护通过专家组评审，永生源铁皮石斛种植基地被授予国家中药现代化科技产业基地和省农业标准化示范区。现代渔业发展加快，水产品总产量17.7万吨、增长5.2%，新认定无公害水产品生产基地6个、水产健康养殖示范场3个、无公害水产品认证12个，深水抗风浪网箱养殖取得显著成效。

农民增收渠道不断增多。全面落实惠农强农政策，全市财政用于“三农”的支出4.94亿元，增长23%。农业龙头企业、重点农产品流通企业等产业化带动能力增强，近10万农户增产增收。供销社系统10多年来首次扭亏为盈，服务“三农”能力进一步提高。农村劳动力培训转移工作扎实开展，技能培训20530人，转移就业56401人。集体林权制度改革积极实施，土地依法流转工作得到推进。“规划到户、责任到人”的扶贫开发工作全面落实，110个贫困村、20424户贫困户获得对口帮扶。

农村发展环境进一步改善。乡村公路建设取得新发展，改造县乡公路39公里，建设通村公路253公里。加快实施水利防灾减灾工程，加固中小型水库70宗、江海堤围45公里，维修渠道218公里，沟尾溪排涝整治工程进展顺利。农村饮水安全工作有效推进，新建成饮水安全工程28宗、新增受益人口42.2万人，饶平沿海六镇饮水解困工程基本完成。新型农村合作医疗继续发展，参合率提高到99.5%。农村养老保险试点工作已经启动，参保人数达到24350人。新农村示范点建设取得新成效。创建林业生态文明村48个。

四、项目带动战略有效实施

投资力度不断加大。全社会固定资产投资163亿元，增长23.1%。其中，第三产业投资73.3亿元，增长32.9%。重点项目建设完成情况良好，53个重点项目完成投资87.2亿元，为年度计划的97.7%。一批项目被纳入上级计划并获得资金支持，其中列入中央新增投资项目63个、省重点建设项目16个。

基础建设扎实推进。基础设施投资76亿元，增长42%。500千伏韩江输变电工程提前竣工。省道231线凤湾线改建、省道334线饶平段大修、仙洲岛堤围整治、安揭引韩灌区节水灌溉改造等工程基本完成。厦深铁路潮州段、汕梅高速公路潮州段、市客运中心枢纽站、城乡电网改造工程、韩江南北堤南堤段达标加固工程等重点项目进展顺利。韩江东西溪大桥奠基建设。

产业园区开发步伐加快。深圳（潮州）产业转移工业园在省竞争性扶持资金竞标中成功中标，获得省5亿元和深圳市2.5亿元资金扶持。园区已有企业80家。潮州投资环境暨深圳（潮州）产业转移园招商推介会在深圳成功举办，签订投资合作项目20个、金额34.3亿元。市径南产业转移园完成大部分征地工作，中国房地产集团公司已进入园区建设。潮州港经济区总体规划及其重化工业园、产业转移园控制性详规编制工作基本完成，进港公路、亚太通用码头、粤东水产品物流中心开工建设。中交集团广州航道有限公司计划投资150亿元，联合开发潮州港公用航道和西澳港区，目前各项前期工作扎实进行。各县区产业园规划建设工作积极推进。

五、市场拉动作用明显增强

文化旅游快速发展。广东省第二届粤东侨博会暨潮州市第三届文化旅游节成功举办，牌坊街、许驸马府修复工程竣工并对外开放，广济桥特种邮票在全国发行，古城文化旅游影响力和吸引力进一步增强。市旅游咨询服务中心挂牌运作，潮州木雕艺术馆建成开放。木雕、潮绣、麦杆画等制作逐步迈向产业化，旅游商品市场日趋活跃。国民旅游休闲计划有效实施，“乡村游集群”列入省旅游重点扶持项目。全市旅游收入46.4亿元，增长32%。

对外贸易稳步回升。及时出台26项扶持措施，支持企业扩大出口、稳住市场，外贸出口从去年6月份开始止跌回升。全市外贸进出口27.88亿美元，增长24.9%。其中，出口18.7亿美元，增长6.5%，增幅居全省各市第6位。陶瓷出口大幅上扬，出口8亿美元，增长33.4%。企业设立境外公司5家，累计8家。新增省出口名牌企业64家，累计85家。

国内市场有效拓展。被商务部确定为全国50个“扩大消费、创新生活”重点活动之一的“广东潮州陶瓷暨特色产品（上海）展销会”成果丰硕，签订经贸合作合同及协议金额82.56亿元。企业开拓市场力度加大，国内销售网络逐步完善，市场占有份额有新增加。规模以上工业内销产值441亿元，增长18.6%。

招商引资成效突出。新批外商投资项目23个，合同利用外资1.44亿美元，增长5.1%。外商增资扩产项目14个，合同增资额1.32亿美元。实际利用外资1.01亿美元，增长11%。招商活动积极开展，粤东侨博会签订合作项目18个，首期投资金额305亿元。区域合作力度加大，融入珠三角经济区和海峡西岸经济区步伐加快，潮台经贸合作进一步升温。

六、城市发展环境更加优化

节能减排工作成效明显。韩江、枫江、黄冈河流域污染整治工作继续推进，水源水质保持稳定。国家环保模范城市创建活动积极开展，创建规划已报请省组织评审。市环境监控中心建成运作，市第一污水处理厂扩建工程完成，饶平县污水处理厂完工，市桥东污水处理厂和潮安县污水处理厂加紧建设。市垃圾卫生填埋场二期工程建成投入使用。节能减排、资源综合利用工作扎实开展，陶瓷清洁生产和窑炉节能技术改造取得成效，全市单位生产总值能耗预计下降3.54%。

资源管理利用得到加强。新一轮土地利用总体规划大纲编制完成，一批重点项目建设用地得到保障。违法违规用地和非法开采矿产资源查处工作进一步加强并取得明显成效，土地管理工作通过国家土地例行督查并受到肯定。利用园地山坡地开发补充耕地工作积极推进，“三旧”改造工作加紧开展，土地资源利用水平不断提高。镇级国土资源所全部挂牌运作，国土管理工作重心下移、关口前移。

城市建设管理力度加大。城市总体规划修编基本完成，镇村规划编制积极进行。金山大桥获得铁道部“优质工程一等奖”。市开发区工业大道、春荣路、新洋路改造工程全面完成，绿榕南路、外环北路和外环西路市区段建设正在推进，潮州大桥奠基建设。三利溪排涝整治一期工程完成，市区积水问题得到缓解。竹竿山水厂扩建工程顺利完成，日供水能力由18万吨提高到33万吨。理顺城市公交管理体制，新增公交线路3条，更换新型公交车72 部，公交事业发展迈上新台阶。城市管理权限下放，层级化责任体系初步形成，脏乱差现象有所改观。我市在国家城市综合整治定量考核中名列全省第5位。

七、各项社会事业全面进步

文明建设继续推进。学习实践科学发展观活动深入开展，新中国成立60周年庆祝活动圆满成功。文明创建活动广泛开展，新时期潮州人精神教育继续推进，未成年人思想道德教育得到加强。

就业保障工作得到加强。社会保障和就业投入5.34亿元，增长8.1%。新增城镇就业30798人，实现再就业13120人，全社会非农就业比重超过74%。城镇登记失业率2.46%，低于全省平均水平。社会保障体系不断完善，城镇养老、医疗、失业和工伤参保人数达到33.4万、33.2万、27.6万和26.5万，分别增长5.7%、57.8%、4.3%和4.4%。养老保险待遇大幅度提高，职工人均每月养老金提高至782元，比上年底增加191元。市直困难国有企业欠缴职工养老保险费问题分三年予以解决，4.5万名困难国有集体企业退休人员得到资助纳入医保范围。最低生活保障覆盖面扩大，低保人数5.98万名，增加0.73万人。新建成农村敬老院12所，集中供养能力有所提高。社会慈善救助活动不断开展，爱心工程惠及人群增多。残疾人就业工作加强，参加技能培训的残疾人1664名。住房解困工作扎实推进，落实廉租住房实物配租74户、租赁补贴796户。对口援建汶川耿达乡工作成效明显，“广东潮州新村”等项目建设进展顺利。

文教体卫全面发展。教育工作成效突出，在省基础教育责任制考核中获得优秀等次。城乡免费义务教育政策得到落实，九年义务教育巩固提高。高中阶段办学规模继续扩大，办学质量不断提升。录用中小学代课教师298人，教师福利待遇逐步提高。市文化艺术中心和市博物馆建成投入使用，市图书馆以及基层文化设施建设扎实推进。非物质文化遗产普查工作完成，新增省级非物质文化遗产保护项目9项、国家级非物质文化遗产传承人3名。全民健身运动广泛开展，市运会圆满成功，市业余体校被确定为“国家高水平体育后备人才基地”。公共卫生体系建设不断完善，中心镇卫生院、社区服务中心配套设施逐步改善。“新农合健康体检、宫颈癌普查”项目全面启动，手足口病和“甲流”防控及时有力。

人口计生等工作水平进一步提高。低生育水平得到保持，人口出生率和自然增长率分别为11.1‰、5.4‰。国防教育、国防建设和“双拥共建”等工作成效明显，审计、司法、外事侨务、统计、打私、广播电视、人防、气象、防震、地方志、档案、老龄、民族宗教等工作取得新发展。

八、管理服务和民主法制加强

行政服务水平不断提高。“落实推进年”活动有效开展，机关效能建设不断深化。第四轮政府机构改革全面实施，医药卫生、财政体制等重点领域改革取得新发展。为民服务全程代理制深受群众欢迎，政府公信力和执行力有新提高。

民主法制建设全面加强。“五五”普法深入实施，行政执法职权规范公开，政府法律顾问制度逐步健全。依法监督、民主监督较好落实，省市49件人大代表建议、101件政协提案全部办结，代表和委员满意和基本满意率100%。与各民主党派、工会、共青团、妇联等联系加强，科学民主决策机制不断完善。政务公开、村务公开深入开展，行政审批电子监察系统进一步健全，政风行风热线节目效果良好。党风廉政建设责任制全面落实，一批违法违纪案件受到严肃查处。

社会保持和谐稳定。治安防控能力提高，刑事发案率总量下降4.2%、破案总量提高12.8%。信访工作进一步加强，群体性事件控制及时、处置得当。安全生产形势稳定，产品质量和食品药品安全水平提高，全年未发生重特大安全事故和食品药品安全事件。应急管理工作继续推进，应急保障能力逐步增强。

各位代表，过去一年取得的成绩，是全市人民共同努力、社会各界大力支持的结果，也得益于我们紧紧地把握好以下几点。一是全力以赴保增长促发展。面对金融危机的冲击，坚决贯彻落实中央和省保增长的决策部署，及时出台促进经济平稳较快发展等一系列文件和规定，在非常时期采取非常措施，统筹利用国际国内两个市场，全力以赴稳定出口扩大内需，加大力度增加投资促进消费，有效地促进经济平稳较快发展。二是突出重点抓调整促升级。在应对危机保增长的同时，利用国际金融危机带来的倒逼压力和转型契机，遵循产业发展和市场经济规律，大力推进产业创新，积极承接产业转移，努力在调整中抓转型促升级，在发展中打基础增后劲。三是毫不动摇保民生促和谐。在财政十分困难的情况下，千方百计增加民生投入，着力推进基本公共服务均等化，解决了一批民生保障和涉及群众切身利益的突出问题，有效地促进了社会和谐稳定。四是坚持不懈抓服务促落实。以深入学习实践科学发展观活动和“落实推进年”活动为动力，切实转变作风，强化责任落实，较好地推动了各项工作的开展。在此，我代表市人民政府，向全市人民、驻潮部队、武警官兵、各民主党派、人民团体和港澳台同胞、海外侨胞、社会各界人士，表示衷心的感谢！

过去一年，我市经济社会发展虽然取得显著成绩，但发展中也存在不少困难和问题，受国内外复杂多变的经济形势影响，又面临着新的挑战和困难。一是经济总量不大，发展水平不高，与发达地区相比差距较大；二是现代产业体系建设步伐不快，产业转型升级较为缓慢，产业核心竞争力不强；三是改善民生任务艰巨，财政增收节支压力较大，科学调控、统筹使用空间较小；四是城市管理存在薄弱环节，社会管理面临新情况新问题，政府工作还存在不足。这些问题，我们将进一步采取措施加以克服解决。

2010年工作安排

今年是实施“十一五”规划的最后一年，也是实现“五年大变化”的关键一年。当前，国际金融市场渐趋稳定，主要经济体的经济有好转迹象，世界经济可望恢复性增长；国内经济回升向好的基础逐步巩固，经济运行积极因素明显增加，企业适应市场变化的能力不断增强。国家保持宏观经济政策的连续性和稳定性，以及省委省政府推动粤东地区“五年大变化”政策措施的落实，为我市经济保持平稳较快发展创造了有利环境和条件。但是，世界经济复苏仍较缓慢，金融危机影响依然存在；国内经济回升内在动力不足，结构性矛盾和问题仍然突出；我市经济发展总体水平偏低，现代产业体系建设滞后。这些问题制约着我市经济社会又好又快发展。因此，我们必须进一步增强加快发展的意识，积极利用好危机中的一切机遇，努力把握好调整中的积极因素，全力化危为机，全面推动经济社会平稳较快发展。

做好今年政府工作，要坚持以科学发展观为指导，全面贯彻落实党的十七大、十七届四中全会和省委十届六次全会、省粤东工作会议、市委十二届八次全会精神，以实现“五年大变化”为目标，以转变经济发展方式为核心，以构建现代产业体系为重点，着力提升产业素质，着力拓展内需空间，着力扩大对外开放，着力优化发展环境，着力保障和改善民生，着力加强政府建设，全面完成“十一五”规划各项目标任务，努力推动经济社会又好又快发展。

综合各方面因素，今年我市经济社会发展的主要预期目标是：生产总值增长13%，单位生产总值能耗下降3.2%，地方财政一般预算收入增长14%，全社会固定资产投资增长20%，外贸出口增长11%，全社会消费品零售总额增长23%，居民消费价格指数涨幅控制在3%以内，人口自然增长率控制在6.5‰以下，城镇登记失业率控制在3%以下，市区居民人均可支配收入增长9%，农村居民人均纯收入增长10%。

为实现上述目标，要扎扎实实抓好以下五个方面工作。

一、调整优化产业结构，全力构建现代产业体系

加快构建现代产业体系，是转变经济发展方式、促进经济又好又快发展的迫切需要。要调整优化产业结构，全力加强产业建设，努力做大做强产业经济。

（一）提升特色产业发展水平

加强创新体系建设。依托现有资源优势和产业基础，积极推进特色产业基地建设，大力发展以龙头企业带动、中小企业集聚为特征的产业集群。加快“中国瓷都创新基地”、产业集群升级示范区、支柱产业共性技术创新中心等服务平台建设，发挥陶瓷、食品、不锈钢等质检机构的作用，推进省水族器材检测站建设，争取国家食品软包装及机械制造、省鞋类产品质检机构获准筹建，进一步建立健全创新服务体系。

推动企业创新发展。落实鼓励创新的政策和规定，完善支持创新的措施和办法，引导创新要素向企业集聚。加快培育一批创新型企业，建设一批省级以上工程中心和企业技术中心，实施一批技术改造项目，推进一批项目积极开展产学研合作。大力推进质量标准化工作，引导企业积极参与各类标准制修订，努力抢占行业“话语权”。继续实施品牌带动战略，做好通花陶瓷、釉下潮彩等地理标志产品申报工作，争创更多的名牌名标。引导企业转变创新观念和方式，促进企业从花式品种创新转向生产方式和提高科技含量创新，奖励引进能够带动行业大发展的新设备新技术，推动陶瓷等传统产业实现新发展。争取相关资金和技术支持，总结推广企业低碳生产经验，积极发展低碳产业项目。

完善产业配套服务。加快培育发展现代服务业，努力发挥现代服务业在促进产业发展中的积极作用。鼓励支持投资产业市场，加快形成陶瓷、纺织服装、不锈钢、鞋类等产品或原材料专业市场，逐步解决有产业无市场的问题。强化金融支撑，推进银企协作，提高金融服务能力。积极发展多层次、多形式信用担保机构，努力解决企业贷款难、担保难问题。鼓励企业融资发展，加强战略投资合作,支持帮助具备条件的企业上市融资，培育储备一批上市后备企业。

壮大产业经济规模。优化资源要素配置，加大投资推动力度，加快形成依托城市、园区的产业集群和产业带。按照“抓大扶强”的原则，加强规模企业培植工作，着力培育一批大型企业集团和行业龙头企业，力争一批企业入选省现代产业“500强”和自主创新“100强”。大力发展民营经济，不断激发全民创业活力，抓好规模以下企业转化工作，进一步做大产业规模。

（二）加快发展壮大港口经济

统筹港口港区发展。严格组织实施港口和经济区总体规划，全面加强岸线、海域、陆域和土地等资源的管理、利用和保护，确保港口及经济区科学有序发展。加强建设用地控制工作，分期分批做好土地征用和储备，确保招商项目能够及时落地建设。加快编制各类控制性详细规划，认真编制港区道路、水电、污水处理等专项规划。围绕建设现代化港口和滨海新区这个目标，尽快编制完善西澳港区发展规划。

推进港口港区建设。加快潮州港进港公路、亚太通用码头、小红山5000吨级多功能货运码头建设，提高港口港区的集疏运能力。积极争取大潮高速公路、厦深铁路进港支线、汕汾高速公路进港延长线获得立项。尽快完成潮州港公用航道及西澳港区综合建设项目前期工作，力争首期工程今年开工建设。争取上级支持和战略合作，力促潮州港供水工程早日动工。

加快临港产业发展。结合国家实施重点产业调整的部署，谋划一批有利于构建现代产业体系的重大项目，争取更多项目列入国家和省计划。协助大唐电厂争取3、4号机组获准送电，力促华丰二期工程、风能发电项目开工建设；大力发展低碳经济，推进普天生物环保城的规划论证并争取相关项目投建。尽快启动潮州港对台直航，积极申报设立海峡两岸商品交易物流中心，提高对台经贸发展水平。

培育发展新兴产业。大力宣传推介潮州港的资源优势和发展前景，提高潮州港的投资吸引力。努力创新招商方式，加强项目策划包装，采取多种形式招商引资，重点引进高新技术、石油化工、仓储物流等重大项目，争取在新技术、新材料、新能源和生物环保、精细化工等新兴产业引进上有所突破。加强协调配合，齐心协力抓服务、促落实。

（三）大力发展文化旅游产业

创新管理经营机制。理顺文化旅游管理体制，实行文物景点、旅游资源统一管理、统筹利用，研究实施古城文化旅游区“一票制”，提高文化旅游经营质量和效果。合理规划文化旅游线路，整合物质和非物质文化旅游资源，建立健全古城牌坊街管理长效机制，增强潮州文化旅游的市场魅力。借鉴国内外旅游发展经验，创新文化旅游经营思路，积极引入投资经营主体参与古城文化旅游区的建设经营。

优化旅游发展环境。加强旅游服务功能建设，改善旅游接待条件，完善旅游指引标识、停车场等设施，推进富丽戴斯、汇景威尼斯等高星级酒店建设。以建设5A级景区为目标，推进实施历史文化名城综合开发项目，尽快完成牌坊街修复工程收尾工作，完善许驸马府陈列配套，启动韩祠橡木园修建工程，做好各类特色街区规划保护，推进旧民居修整利用，加快海外潮人博物馆建设，完善古城文化旅游区各项配套设施。重视旅游从业人员培训管理，规范旅游市场秩序，营造良好的旅游发展环境。

提升宣传推介水平。突破传统思维模式，创新旅游宣传推介方法，加强旅游整体形象策划，大力宣传以广济桥和牌坊街为亮点、以古城文化旅游区为主体的潮州特色文化旅游，加快把潮州文化旅游打造成为国内外知名品牌。积极组织参加各类旅游推介活动，大力开展各种主题宣传，重视发挥星级酒店、旅行社、导游在旅游促销中的作用，全面提升宣传促销效果。

壮大旅游产业规模。促进旅游吃、住、行、游、购、娱等要素协调发展，努力实现旅游经济效益最大化。扶持发展传统工艺美术产业，积极培育发展旅游制造业。加快广东（潮州）旅游商品基地建设，提高旅游商品市场发展水平。以文化旅游为龙头，逐步发展工业、会展、商务及科普旅游等，进一步做大做强旅游产业。

（四）扎实发展农业农村经济

加快特色农业发展。积极实施科技兴农战略，努力稳定粮食生产，提升茶叶、水果、水产、花卉等特色农业发展水平。成立潮州市茶叶科研中心，提高茶叶基地化、标准化、产业化水平。发挥农业龙头企业、供销社系统、流通组织的作用，加强农产品质量认证和品牌建设，推进特色农业规模化发展。规划建设好现代渔港经济区，大力发展深水抗风浪网箱养殖，促进现代渔业加快发展。

提高农业农村发展能力。搞好农田水利建设，加强现代标准农田、大中型灌区及节水改造工程建设，改善农业生产条件。启动自然村公路硬底化工程，加强农村污水、垃圾处理设施和饮水安全工程等建设，搞好农村环境综合治理，改善农村发展条件。推进集体林权制度改革，促进林业发展、林农增收。支持农村土地承包经营权依法流转，鼓励农民采取出租、入股、置换等多种方式发展适度规模经营。加大扶贫开发力度，确保“规划到户、责任到人”工作全面落实。

努力促进农民增收。实施惠农强农政策，落实农业各项补贴，加大对“三农”的投入。推进农村劳动力培训和转移就业，鼓励引导外出务工农民回乡创业。挖掘农业内部增收潜力，引导农民开展生态、休闲、观光等农业经营活动，千方百计增加农民收入。

二、加强投资与市场开拓，促进经济平稳较快发展

扩大投资、促进出口和消费，是保持经济平稳较快发展最直接、最有效的办法，要继续加大力度抓紧抓好。

（一）努力促进投资有效增长

抓好重大项目规划储备。结合“十二五”规划的编制，认真谋划一批关系全局和未来发展的重大项目，研究提出涉及交通、能源、水利、卫生、教育、环保、市政等方面的项目，进一步充实市重点项目库。按照“兼顾全面、突出重点”的原则，统筹优化重大项目布局，对于符合国家产业导向、前期工作成熟的重大项目，积极申报立项并争取上级支持。

推进一批重点项目建设。认真抓好今年计划实施的54个重点项目，确保完成年度90.1亿元的投资目标。交通方面，着力抓好韩江东西溪大桥建设，配合支持厦深铁路和汕梅高速公路潮州段建设，争取潮揭高速公路尽快复建，抓好漳玉高速公路潮州段建设前期工作，落实潮汕火车站进站公路建设计划，抓好省道233线枫溪至彩塘路段大修、国道324线饶平段中修等工程。水利方面，积极推进潮州供水枢纽配套工程建设，全面完成韩江南北堤南堤段、黄冈河大堤和中小型水库除险加固工程，力争沟尾溪、内洋南总干一期等排涝工程建成，确保河内湖排涝整治工程开工建设。能源方面，争取在建2座220千伏输变电工程上半年建成投产，庵埠220千伏输变电工程开工建设。

努力促进多元化投融资。抢抓中央保持投资适度增长的时机，以国债资金、中央和省专项资金为重点，努力争取更多的资金支持。进一步创新理念，扩大与国家开发银行的合作，采取BT、BOT等形式融入建设资金。加快金山投资公司、潮发担保公司和韩江投资公司等投融资平台建设，编制民间资本投资产业指南和重点项目计划，积极引导和鼓励社会投资，促进社会投资与政府投资共同发展，推动固定资产投资不断增长。

（二）积极推动内外经贸发展

大力拓展国内市场。积极组织实施“潮货北上西行”，筹划在国内重要城市举办潮州特色产品展销会，继续组织企业参加各类展销活动，努力提高“潮货”的市场占有率和企业品牌影响力。推广企业设立直销店、建立代理制等成功经验，帮助支持企业设立营销网络、抢占国内市场。加快集贸市场、批发市场建设，推进“万村千乡”市场工程、家电汽车下乡和“以旧换新”等工作，全面激发城乡消费活力。

积极发展对外贸易。继续用好外贸出口扶持政策，帮助企业申请各类专项扶持资金。坚持科技兴贸，不断推进创新型企业和出口生产基地建设，引导鼓励企业打造自主出口品牌。深入实施市场多元化战略，努力稳定传统市场，积极开拓新兴市场，促进外贸出口稳步增长。紧紧把握好中国－东盟自由贸易区正式建立并实行零关税的机遇，采取措施帮助企业大力开拓东盟市场。针对贸易保护主义常态化并将长期影响出口的实际，引导企业增强风险意识和危机意识，积极主动应对各种挑战。进一步完善通关条件，优化口岸管理工作，合力营造良好的外贸发展环境。

努力加强招商引资。全方位、多形式宣传推介我市的发展优势和投资环境，不断增强潮州的投资吸引力。发挥港口、产业等优势，强化投资载体建设，创新招商引资方式，争取在重大项目引进上有新突破。加强与潮籍商会、潮籍乡亲的联络，促进“以侨引资”和“乡贤回归”工程实施。着力扩大区域交流合作，积极承接发达地区产业和技术转移。重视对台招商引资，加快规划和申报国家级台商投资区。切实加强跟踪服务工作，重点抓好侨博会等一批签约项目的落实。

三、重视“双化”互动发展，努力提升城市发展水平

坚持工业化带动城市化、城市化促进工业化的发展方向，针对我市城市化水平偏低的实际，全力以赴加快城市化进程。

（一）不断优化城市发展环境

拓展城市发展空间。抓紧完成《潮州市城市总体规划（2006－2020）》的报批工作，按照“完善西部、拓展东部、扩大空间、带动周边”的思路，加紧推进潮州大桥建设，全力推动城市向东扩展。尽快编制韩江东岸溪南片、溪北片、意东综合片等重点区域的控制性详细规划，严格各类规划的实施，坚决查处违反城乡规划的建设行为。

推进城市基础建设。配合城市向东扩展发展战略的实施，加快外环北路、外环西路、银槐北路等道路建设，尽快完成绿榕南路等工程，不断改善城市交通环境。制订城市排涝规划并分步实施，逐步解决城市排涝问题。加强市区西片和桥东片排污管网建设，增强城市污染防治能力。加强城市园林景观建设，完善城市公共设施和服务功能，把城市做精、做优、做美。

全面加强城市管理。理顺城市管理体制，落实城市管理责任，下放城市管理权限，形成责任明确的层级管理网络。加大宣传力度，引导市民支持和参与城市管理。大力整治占道经营和沿街摆卖行为，整治交通违法违规行为，整治城乡结合部脏乱差现象，推进道路保洁机械化作业，提高城市美化净化水平。大力发展城市公共交通，完善公交线路和站点的设置；加强城市交通组织，加快停车场所的规划建设，整治车辆乱停乱放，努力改善城市交通环境。

（二）积极实施“双转移”战略

加强产业园区建设。大力推进径南产业转移园各项基础设施建设，加快提升临港产业转移园发展水平，引进和转移一批企业在园区集聚发展。完善潮州经济开发区产业转移园的设施和功能，争取把该园区申报为省级高新技术产业区。扩大与深圳等珠三角地区的联系合作，积极承接发达地区产业转移。认真搞好潮安特色产业基地、饶平望海岭产业园、湘桥河内特色工艺创意园、枫溪陶瓷创意园等工业园区的论证、规划和建设，引导企业向园区集聚发展，把工业园区建设成为城市工业新区。

促进农村劳动力转移。发挥城市对农村的带动辐射作用，推动城乡协调发展。把解决农村转移人口在城市就业和落户，作为推进城市化发展的重要工作认真抓好。推进户籍制度改革，引导符合条件的农村转移人口落户城市，加强农村劳动力技能培训和转移就业工作，促进劳动力跨城乡合理流动，为工业化提供必要的劳动力资源。

（三）全面提高发展承载能力

加强环境生态保护。推进国家环保模范城市创建工作，加快实施创模重点工程。加强饮用水源水质保护，确保群众饮水安全。严格执行环境影响评价制度和项目建设“三同时”制度，努力控制主要污染物新增排放量。加大执法力度，严肃查处超标排污等环境违法行为。重视海洋、湿地、林地和地质环境保护，推进生态公益林和沿海防护林等生态工程建设。

狠抓节能减排工作。大力发展循环经济，搞好资源综合利用和清洁生产，建设陶瓷废渣回收利用示范工程。完善节能标准体系和计量保证体系，督促高耗能企业加强计量器具配置，推进以陶瓷窑炉为主的节能降耗技术改造。尽快完成市桥东污水处理厂和潮安污水处理厂建设，确保全年节能减排目标实现。

推进节约集约用地。加快旧城镇、旧厂房、旧村庄改造步伐，加大闲置土地处置力度，努力提高土地利用率。严格控制新增建设用地占用耕地，保障重点项目和基础设施建设的合理用地需求。落实土地管理共同责任制，坚决查处土地违法违规行为。积极利用园地、山坡地开发补充耕地，确保全市耕地占补平衡。

强化矿产资源管理。加快第二轮矿产资源总体规划编制工作，优化矿产资源开发布局。整顿矿产资源开发秩序，规范采矿权审批管理，坚决打击非法开采稀土、瓷土和河砂等行为。加强地质灾害防治工作，保护人民群众生命财产安全。

四、全面发展社会事业，着力保障和改善民生

实现社会和谐稳定、百姓安居乐业，是政府的重要职责。要积极采取措施，促进各项社会事业发展，进一步保障和改善民生。

加大公共财政投入。加强税收征管制度建设，完善综合治税网络，落实征管责任，强化控管措施，确保各项收入足额入库。调整优化支出结构，保障重点支出需要，加大对民生领域和社会事业的支持保障力度，促进基本公共服务均等化。继续做好援建汶川耿达乡工作。

加强各项民生工作。大力实施保障性安居工程，落实廉租房210套、租赁补贴140户，努力解决困难群众住房难问题。推进饮水安全工程建设，争取未开工项目上半年全部开工。加强排涝渠系治本工作，彻底根治市区内涝问题。加快客货运输站场规划建设，全面提升城乡交通服务水平。

推进城乡统筹就业。完善城乡公共就业服务体系，促进高校毕业生、农民工、就业困难人员实现就业再就业；鼓励自主创业、自谋职业，推动以创业带动就业，力争城镇新增就业2.5万人。落实全民技能提升储备计划，加快技工教育发展，加强职业技能培训，提高劳动者素质。

完善社会保障体系。健全城镇职工基本养老保险、基本医疗保险等制度，强化养老保险扩面征缴工作，扩大各类保险覆盖面。按计划解决市直困难国有企业欠缴职工养老保险费问题，积极推进新型农村养老保险试点。完善新型农村合作医疗制度，争取更多城镇居民参加基本医疗保险，探索建立城乡一体化医疗保障制度。加强城乡低保、五保供养、救灾救济等工作，保障城乡困难群众基本生活。推进扶贫帮困工作，努力解决特困群众看病、住房、子女上学等困难。认真落实相关政策，解决好优抚对象、企业军转干部、伤残军人等特殊群体的生活问题。

促进教育事业发展。扩大优质教育资源，鼓励民间资本办学，推进教育均衡发展。深入实施中小学校舍安全工程，加强义务教育规范化学校建设。扩建一批高中阶段学校，大力发展职业教育，完成市职校新校区建设，力争高中阶段毛入学率达到80%。解决好中小学代课教师问题，落实教师工资福利待遇“两相当”。

提高文化体育发展水平。完善公共文化服务体系，加快市图书馆、市档案馆建设，办好基层文化站，新建一批农家书屋，组织文化下乡和电影下乡，丰富城乡群众文化生活；加强非物质文化遗产保护、传承和利用，积极做好申报国家级潮州文化生态保护区工作。发挥史志资政作用，加快第二轮新编地方志进度。做好市体育中心的选址规划，大力开展群众性体育活动。

推进卫生人口计生工作。深化医药卫生体制改革，构筑三级医疗卫生服务网络；做好市中心医院、中医院、妇幼保健院、精神病院异地新建前期工作，加强社区卫生服务机构和乡镇卫生院建设；做好“甲流”等疾病的防控工作，确保人民群众生命健康。规范流动人口管理服务，稳定低生育水平，力争人口计生工作进入全省先进行列。

维护社会和谐稳定。弘扬新时期潮州人精神，广泛开展文明创建活动。加强普法工作，全面完成“五·五”普法任务。重视做好信访工作，积极化解各类矛盾，维护群众合法权益。加强社会治安综合治理，依法严厉打击刑事犯罪活动。狠抓安全生产工作，严防各类安全事故发生。扎实推进食品药品安全整治，全力保障人民群众饮食用药安全。强化应急基础建设，提高应急保障和处置能力。

统筹发展各项社会事业。全面做好人才、民族宗教、广播电视、新闻出版、外事侨务、对台、人防、气象、防震、统计、物价、档案、老龄、残疾人、国防和民兵预备役等各项工作。

五、加强政府自身建设，提高驾驭发展全局能力

紧紧围绕促进经济社会又好又快发展这个目标，全面加强政府自身建设，进一步提高依法行政和公共服务水平。

科学谋划发展。树立世界眼光，加强战略研究，坚持科学发展、民生优先，以构建现代产业体系、加快转变发展方式为重点，高质量、高水平编制“十二五”规划。

坚持依法行政。自觉接受人大及其常委会的监督和政协民主监督，认真办理人大代表建议和政协委员提案，主动听取各民主党派、工商联、无党派人士和各人民团体的意见建议。扩大基层民主，完善政务、村务公开。建立健全重大决策规则和程序，严格按照行政程序和规定行使职权、履行职责。落实行政执法责任制、过错责任追究制，全面规范执法行为。

推进机构改革。坚持以改革激发活力，全面组织实施政府机构改革，优化组织结构，理顺部门职责，完善运行机制。推进事业单位分类管理等各项改革，提高政府工作整体效能。

转变政府职能。创新政府管理理念和服务手段，加快服务政府、责任政府、法治政府、廉洁政府和效能政府建设。深化行政审批制度改革，加强各级行政服务中心建设，优化“一站式”行政服务，完善为民服务全程代理制，提高行政效率和服务水平。强化公共服务和社会管理职能，维护社会公平公正。

改进工作作风。健全目标责任管理制度，加大督查工作力度，形成雷厉风行、令行禁止的风气，确保政府各项工作落到实处。大力弘扬求真务实之风，深入基层、深入群众，加强调查研究，切实为群众解难题、办实事、谋福祉。

加强廉政建设。坚持为民、务实、清廉，着力完善惩治和预防腐败体系，全面落实党风廉政建设责任制。强化监察和审计工作，建立健全行政监督机制，严肃查处各种腐败行为。加强公务员队伍建设和管理，努力打造一支高素质、专业化的公务员队伍。

各位代表：形势催人奋进，发展刻不容缓。让我们在省委、省政府和市委的正确领导下，深入贯彻落实科学发展观，振奋精神、锐意进取，脚踏实地、扎实工作，为推动潮州加快发展、科学发展而不懈努力！
